# Phosphate-Catalyzed Succinimide Formation from Asp Residues: A Computational Study of the Mechanism

**DOI:** 10.3390/ijms19020637

**Published:** 2018-02-24

**Authors:** Ryota Kirikoshi, Noriyoshi Manabe, Ohgi Takahashi

**Affiliations:** Faculty of Pharmaceutical Sciences, Tohoku Medical and Pharmaceutical University, 4-4-1 Komatsushima, Aoba-ku, Sendai 981-8558, Japan; kirikoshi@tohoku-mpu.ac.jp (R.K.); manabe@tohoku-mpu.ac.jp (N.M.)

**Keywords:** aspartic acid residue, succinimide, isomerization, racemization, non-enzymatic reaction, buffer catalysis, dihydrogen phosphate ion, proton transfer, computational chemistry, density functional theory

## Abstract

Aspartic acid (Asp) residues in proteins and peptides are prone to the non-enzymatic reactions that give biologically uncommon l-β-Asp, d-Asp, and d-β-Asp residues via the cyclic succinimide intermediate (aminosuccinyl residue, Suc). These abnormal Asp residues are known to have relevance to aging and pathologies. Despite being non-enzymatic, the Suc formation is thought to require a catalyst under physiological conditions. In this study, we computationally investigated the mechanism of the Suc formation from Asp residues that were catalyzed by the dihydrogen phosphate ion, H_2_PO_4_^−^. We used Ac–l-Asp–NHMe (Ac = acetyl, NHMe = methylamino) as a model compound. The H_2_PO_4_^−^ ion (as a catalyst) and two explicit water molecules (as solvent molecules stabilizing the negative charge) were included in the calculations. All of the calculations were performed by density functional theory with the B3LYP functional. We revealed a phosphate-catalyzed two-step mechanism (cyclization–dehydration) of the Suc formation, where the first step is predicted to be rate-determining. In both steps, the reaction involved a proton relay mediated by the H_2_PO_4_^−^ ion. The calculated activation barrier for this mechanism (100.3 kJ mol^−1^) is in reasonable agreement with an experimental activation energy (107 kJ mol^−1^) for the Suc formation from an Asp-containing peptide in a phosphate buffer, supporting the catalytic mechanism of the H_2_PO_4_^−^ ion that is revealed in this study.

## 1. Introduction

There are optical isomers of l- and d-forms for amino acids. All of the amino acid residues that constitute proteins in vivo had been considered to be the l-form, except for glycine (Gly). Recently, however, it has become clear that d-amino acid residues are also widely present in proteins and peptides. Biologically uncommon d-amino acid residues are known to be related to aging, and have been found in various tissues such as those in the lens [1,2,3,4], tooth [5], aorta [6], brain [7,8], bone [9], and skin [10,11] of elderly donors. Moreover, their relationships with age-related pathologies such as cataract and Alzheimer’s disease have also attracted considerable attention [4,8,12,13,14,15,16]. 

**Scheme 1:**
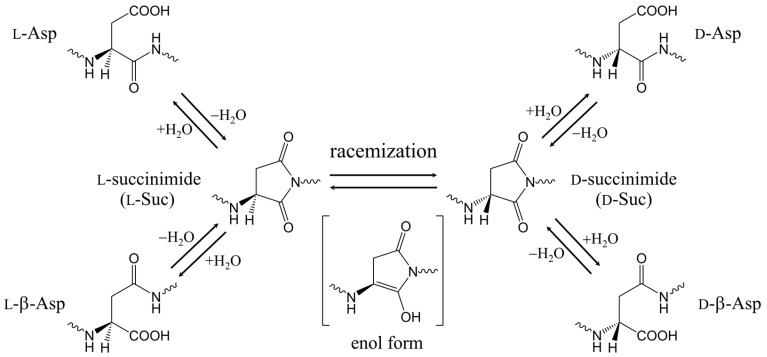
Isomerization and racemization of Aspartic acid (Asp) residues via the five-membered ring succinimide intermediate (aminosuccinyl residue, Suc). A total of four isomers are generated. At around pH 7, the Suc racemization is expected to occur through an enol intermediate.

Most of the amino acid residues that are prone to racemization are aspartic acid (Asp) residues. This is because the racemization occurs easily via the five-membered ring succinimide intermediate (aminosuccinyl residue, Suc) (Scheme 1) [17,18,19]. In this mechanism, the actual species undergoing racemization is Suc, and due to the difference in the position of the ring-opening, a total of four isomers can be produced, i.e., l-Asp and l-β-Asp from l-Suc, and d-Asp and d-β-Asp from d-Suc. For peptides, a typical ratio of l-Asp and l-β-Asp (or d-Asp and d-β-Asp) residues is 1:3 [17,18,20,21,22]. Therefore, the susceptibility to the Suc formation is reflected by the isomerized and/or racemized Asp residues.

**Scheme 2:**
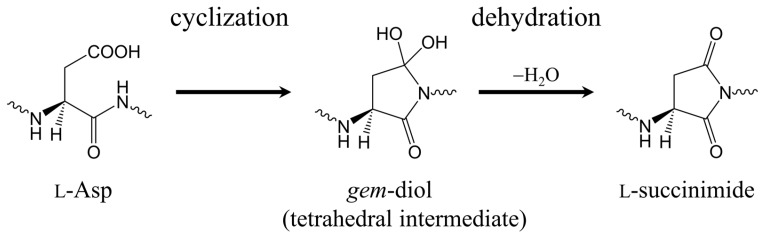
Two-step (cyclization–dehydration) mechanism for the succinimide formation from an Asp residue. In the first step, the nucleophilic attack gives rise to a *gem*-diol (tetrahedral intermediate). In the second step, a water molecule is released from this intermediate to give the succinimide species.

The Suc residue is formed by the nucleophilic attack of the main chain nitrogen of the C-terminal adjacent residue on the Asp carboxyl carbon, with the release of a water molecule. This is an intramolecular nucleophilic substitution reaction, and is basically regarded to proceed in two steps (cyclization–dehydration, Scheme 2) [22,23].

Although the above reactions via the succinimide intermediate in proteins and peptides are non-enzymatic, they will not occur without a catalyst at room or physiological temperature because of high activation barriers [24,25]. We have previously shown that the Asp cyclization can be catalyzed by water or acetic acid molecules by density functional theory (DFT) calculations [23,25,26]; however, what the actual catalysts are in vivo has not yet been clarified.

The experiment by Tomizawa et al. [27] on the hen egg-white lysozyme suggests that Asp racemization is accelerated by phosphate ions at pH 6. At this pH, phosphate ions exist mainly as dihydrogen phosphate, H_2_PO_4_^−^ (p*K*_a_ 6.82 [28]). In vivo, this ion is also present in a significant amount (about one fifth of the total phosphate species), and plays an important role in maintaining homeostasis. We have recently shown by DFT calculations that the racemization of the succinimide intermediate via an enol intermediate (the central part of Scheme 1) is effectively catalyzed by a H_2_PO_4_^−^ ion [29]. Thus, the H_2_PO_4_^−^ ion was proposed to be a candidate for an in vivo catalyst of the succinimide racemization. This success motivated us to investigate the possibility that this ion may also catalyze the Asp cyclization reaction to yield a Suc residue. Very recently, Nakayoshi et al. [30] reported a DFT study on the Suc formation from Ac–d-Asp–Gly–NHMe catalyzed by a H_2_PO_4_^−^ ion. However, an unnaturally large conformational change was involved in the reaction pathway reported.

**Figure 1:**
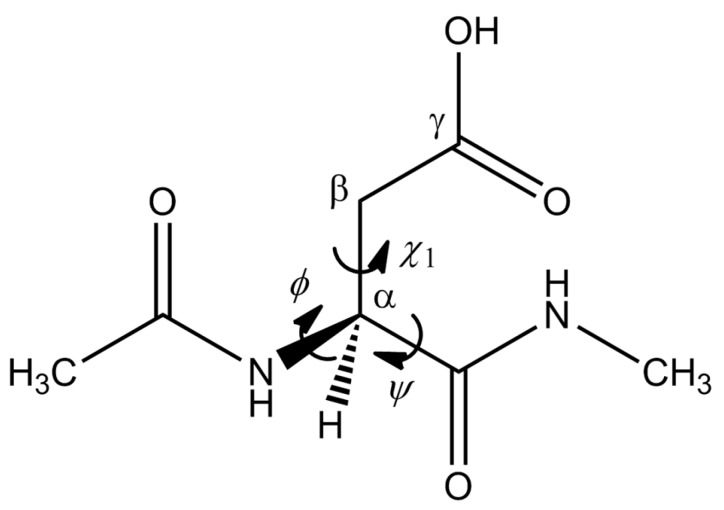
The model compound used in the present study (Ac–l-Asp–NHMe, Ac = acetyl, Asp = aspartyl, and NHMe = methylamino). The *φ* (C–N–C_α_–C) and *ψ* (N–C_α_–C–N) dihedral angles that characterize the main-chain conformation, and the *χ*_1_ dihedral angle (N–C_α_–C_β_–C_γ_) that characterizes the side-chain conformation are indicated.

In the present paper, we focus on the Suc formation from Asp residues, especially on the catalytic role of the H_2_PO_4_^−^ ion. We employed the B3LYP DFT method, as in the previous study on the Suc racemization [29]. The compound shown in Figure 1, in which an l-Asp residue is capped with acetyl (Ac) and methylamino (NHMe) groups on the N and C termini, respectively (Ac–l-Asp–NHMe), was used for the present study. The succinimide product from this model compound is the same as the Suc-containing compound used in the previous study [29]. As in this previous study, only the extended main chain conformation was considered. A H_2_PO_4_^−^ ion (as a catalyst) and two explicit H_2_O molecules (as solvent molecules stabilizing the negative charge) were included in the calculations. Kiriukhin and Collins [31] estimated the “dynamic hydration numbers (ADHNs)” for biologically important ions by using size-exclusion chromatography. The ADHN for an ion was interpreted as the number of tightly bound water molecules that move with the ion. For H_2_PO_4_^−^, the ADHN was estimated to be 1.91 ± 0.17. This may support the validity of the two water molecules included in the present calculations.

## 2. Results and Discussion

**Figure 2:**
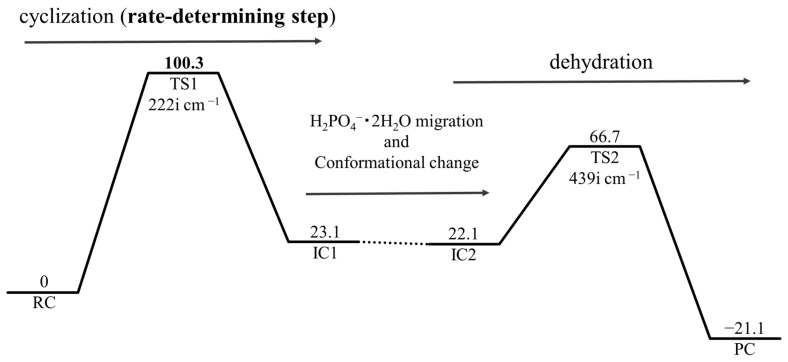
Energy profile for the two-step succinimide formation catalyzed by the H_2_PO_4_^−^ ion. RC: reactant complex; TS: transition state; IC: intermediate complex; PC: product complex. The relative energies after the zero-point energy (ZPE), thermodynamic (25 °C), and hydration Gibbs energy corrections are shown in kJ mol^−1^ with respect to the RC. Also shown are the imaginary frequencies of the two transition states.

**Figure 3:**
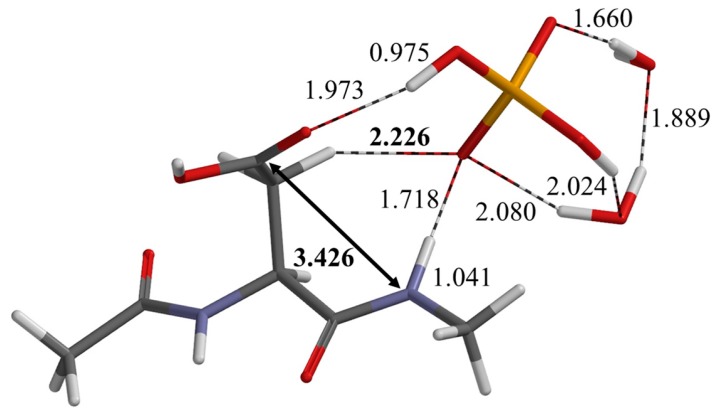
The geometry of the reactant complex (RC). This complex is formed between the model compound (Figure 1), a H_2_PO_4_^−^ ion, and two water molecules. Selected interatomic distances are shown in Å. Grey: carbon; white: hydrogen; blue: nitrogen; red: oxygen; orange: phosphorus.

**Figure 4:**
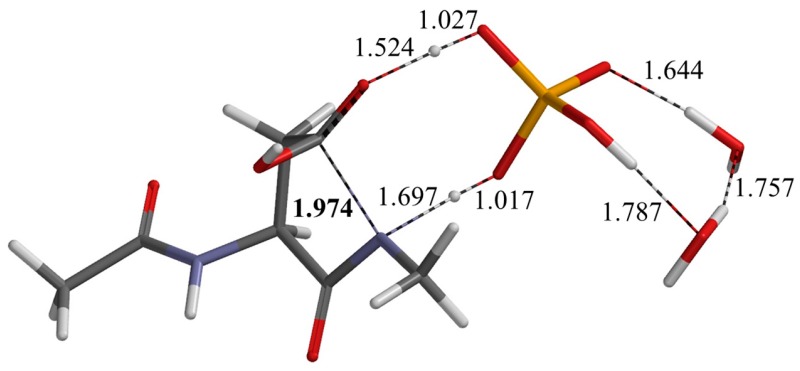
The geometry of the first-step transition state (TS1). This transition state is for cyclization, and connects the RC (Figure 3) and the first tetrahedral intermediate complex (IC1) (Figure 5). Selected interatomic distances are shown in Å. Grey: carbon; white: hydrogen; blue: nitrogen; red: oxygen; orange: phosphorus.

**Figure 5:**
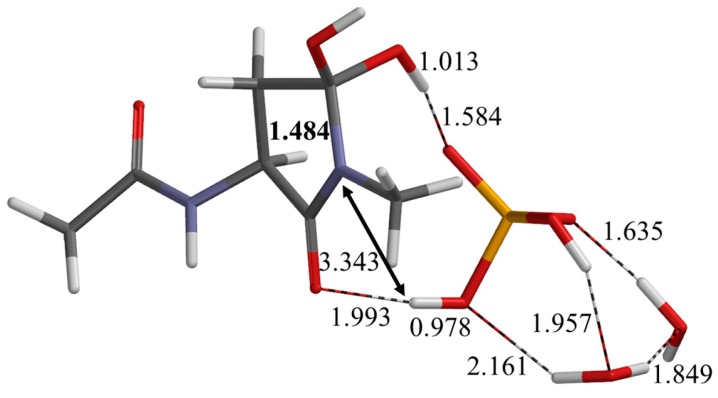
The geometry of the intermediate complex IC1. This complex is directly connected to TS1 (Figure 4). Selected interatomic distances are shown in Å. Grey: carbon; white: hydrogen; blue: nitrogen; red: oxygen; orange: phosphorus.

**Figure 6:**
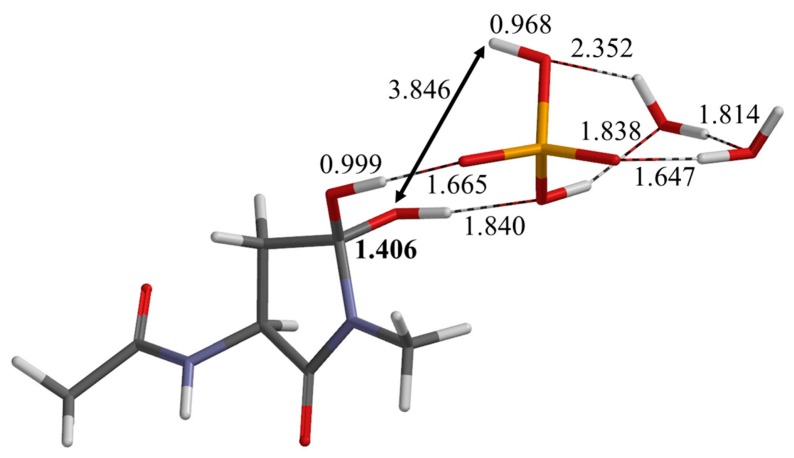
The geometry of the intermediate complex IC2. This complex is directly connected to TS2 (Figure 7). Selected interatomic distances are shown in Å. Grey: carbon; white: hydrogen; blue: nitrogen; red: oxygen; orange: phosphorus.

**Figure 7:**
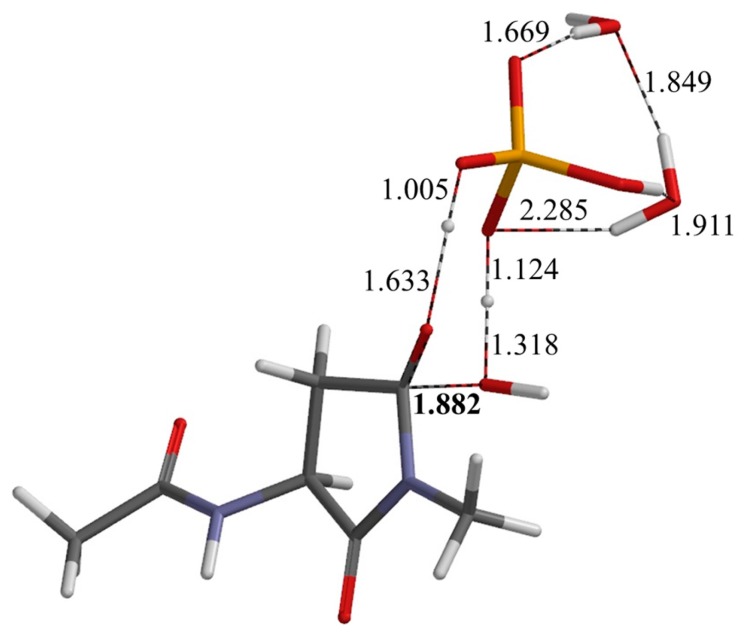
The geometry of the second-step transition state (TS2). This transition state is for dehydration, and connects IC2 (Figure 6) and the PC (Figure 8). Selected interatomic distances are shown in Å. Grey: carbon; white: hydrogen; blue: nitrogen; red: oxygen; orange: phosphorus.

**Figure 8:**
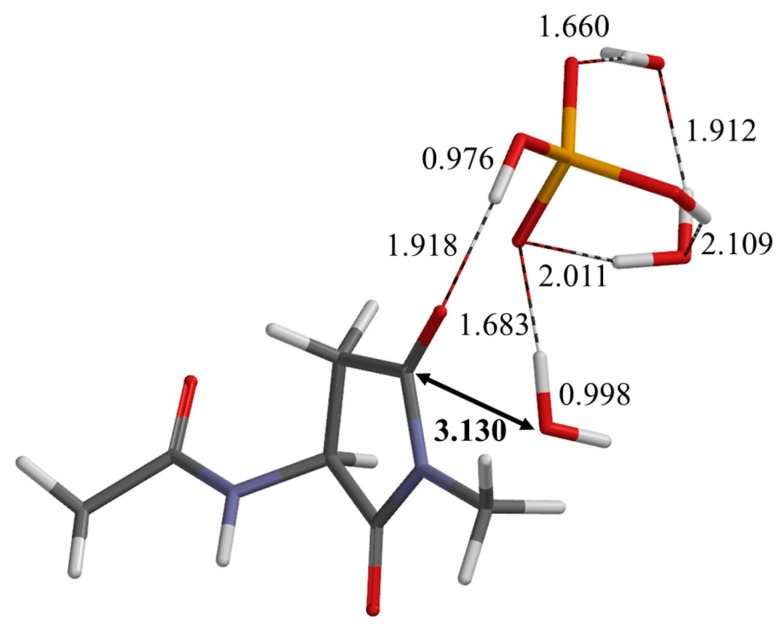
The geometry of the product complex (PC). This complex is directly connected to TS2 (Figure 7). Selected interatomic distances are shown in Å. Grey: carbon; white: hydrogen; blue: nitrogen; red: oxygen; orange: phosphorus.

Figure 2 shows the energy profile in water for the two-step succinimide formation catalyzed by the H_2_PO_4_^−^ ion obtained in this study, and Figure 3, Figure 4, Figure 5, Figure 6, Figure 7 and Figure 8 shows the optimized geometries. RC is the reactant complex consisting of the model compound and H_2_PO_4_^−^∙2H_2_O. PC is the product complex consisting of Ac–l-Suc–NMe and H_2_PO_4_^−^∙3H_2_O. Further, TS1 and TS2 are the transition states of the first step (cyclization) and the second step (dehydration), respectively. IC1 and IC2 are the tetrahedral intermediate complexes directly connected to TS1 and TS2, respectively, and are different from each other in the conformation of the *gem*-diol moiety and the position of H_2_PO_4_^−^∙2H_2_O. Geometry optimizations were performed in the gas phase by the DFT method using the B3LYP functional and the 6-31+G(d,p) basis set. Furthermore, single-point calculations of hydration Gibbs energies were carried out for the gas-phase optimized geometries by the SM8 (solvation model 8) continuum model [32,33] using the 6-31G(d) basis set. The relative energies shown in Figure 2 are those after the zero-point energy (ZPE), thermodynamic (25 °C), and hydration Gibbs energy corrections. The dihedral angles of the optimized geometries defined in Figure 1 are shown in Table 1; the Cartesian coordinates, total energies, ZPEs, and hydration Gibbs energies of the optimized geometries are provided in Tables S1–S7. 

### 2.1. First Step: Cyclization

Figure 3 shows the optimized geometry of the reactant complex (RC) formed between Ac–l-Asp–NHMe and H_2_PO_4_^−^∙2H_2_O. The structure of the H_2_PO_4_^−^∙2H_2_O moiety in RC is the optimal one among those found in our systematic search. In the RC, two hydrogen bonds and one CH–O interaction are observed between Ac–l-Asp–NHMe and the H_2_PO_4_^−^ ion. These two hydrogen bonds are between the Asp side-chain carbonyl oxygen and H_2_PO_4_^−^ (1.973 Å), and between the NH proton of NHMe and H_2_PO_4_^−^ (1.718 Å), and are important as they are involved in the proton transfers occurring in the first step (see below). The CH–O interaction is observed between one of the two Asp β-protons and H_2_PO_4_^−^ (2.226 Å, indicated by boldface in Figure 3), and should have an additional contribution in stabilizing RC. In addition, the H_2_PO_4_^−^ ion and the two water molecules are bound to each other by four hydrogen bonds (1.660, 1.889, 2.024, and 2.080 Å). In the RC, the distance between the carbon (C_γ_) and nitrogen atoms to form a bond during the ring closure is 3.426 Å (indicated by boldface in Figure 3).

Figure 4 shows the transition state TS1 (transition state of the first step) connecting the RC and the IC1 (complex of the tetrahedral intermediate and H_2_PO_4_^−^·2H_2_O directly connected to TS1, Figure 5). The activation barrier of the first step (cyclization forming the tetrahedral intermediate) was calculated to be 100.3 kJ mol^−1^. The energy of TS1 is higher than that of TS2 (transition state of the second step) by about 34 kJ mol^−1^, indicating that the first step (cyclization) is rate determining. Experimentally, an Arrhenius plot was reported by Aki et al. [19] for the succinimide formation from an Asp-containing peptide in phosphate-buffered saline (PBS, pH 7.4). From the slope of this plot, the activation energy is calculated to be 107 kJ mol^−1^. Our calculated barrier (100.3 kJ mol^−1^) is in reasonable agreement with this experimental value.

In TS1, the abstraction of the NH proton by the oxygen atom of H_2_PO_4_^−^, which originally formed the hydrogen bond to this proton, is almost completed (the NH and OH distances are 1.697 and 1.017 Å, respectively). Therefore, the nucleophilicity of the peptide nitrogen is enhanced in an early stage of the first step (note that the amide nitrogen is generally poorly nucleophilic). The distance to the carbonyl carbon atom (C_γ_) of the Asp side chain is drastically shortened to 1.974 Å in TS1 (indicated by boldface in Figure 4), indicating that a bond is being formed between these atoms. Additionally, the proton transfer from H_2_PO_4_^−^ to the Asp side-chain carbonyl oxygen is in an initial stage, and a H_3_PO_4_-like moiety is temporarily created in TS1. Due to cyclization, relatively large changes occur in *ψ* and *χ*_1_ (by 38° and 29°, respectively) when going from the RC to TS1, while *φ* is almost unchanged (see Table 1).

IC1, as shown in Figure 5, is the intermediate complex that is directly connected to TS1, and is composed of the tetrahedral intermediate and H_2_PO_4_^−^·2H_2_O. The energy of IC1 relative to the RC is 23.1 kJ mol^−1^. The proton transfer from H_2_PO_4_^−^ to the Asp side-chain carbonyl oxygen has been completed (the OH distances are 1.013 and 1.584 Å). The distance of the newly formed C–N bond is 1.484 Å (indicated by boldface in Figure 5). A relatively large change in *χ*_1_ (by 41°) was observed in going from TS1 to IC1.

There are three important changes in the first step: (i) the proton transfer from the NH to H_2_PO_4_^−^, (ii) the nucleophilic attack of the “deprotonated” nitrogen on the Asp C_γ_ atom, and (iii) the proton transfer from H_2_PO_4_^−^ to the Asp side-chain. These three changes occur in a domino-like manner. Change (i) enhances the nucleophilicity of the nitrogen atom and induces change (ii), which, in turn, accumulates a negative charge on the side-chain carbonyl oxygen, inducing change (iii), i.e., the second proton transfer. In Figure S1, we provided four representative geometries on the intrinsic reaction coordinate (IRC) of the first step to show the domino-like changes.

### 2.2. Second Step: Dehydration

Figure 6 shows the intermediate complex IC2 directly connected to TS2. The position of H_2_PO_4_^−^·2H_2_O and the conformation in the *gem*-diol moiety in IC2 are different from those in IC1. Both of the diol protons form a hydrogen bond to the H_2_PO_4_^−^ ion (1.665 and 1.840 Å). The relative energy of IC2 with respect to RC is 22.1 kJ mol^−1^, which is about 1 kJ mol^−1^ lower than IC1. The conversion of IC1 to IC2 is expected to occur without a high energy barrier.

Figure 7 shows the second-step transition state TS2 connecting IC2 and the PC (complex of Ac–l-Suc–NMe and H_2_PO_4_^−^∙3H_2_O directly connected to TS2, as shown in Figure 8). The local activation barrier for this second step of the succinimide formation was about 44.6 kJ mol^−1^, which is much lower than in the first step (cyclization). The abstraction of one of the diol OH protons by the H_2_PO_4_^−^ ion is almost completed, as may be seen from two OH distances, 1.633 and 1.005 Å, as shown in Figure 7. That is, a H_3_PO_4_-like moiety is also temporarily formed in the second step. Also, the other OH group of the *gem*-diol moiety is leaving; that is, the corresponding C–O bond is being broken (1.882 Å, indicated by boldface in Figure 7). This distance was 1.406 Å in IC2 (indicated by boldface in Figure 6). Furthermore, a proton transfer from H_2_PO_4_^−^ to the leaving oxygen atom has already started for the formation of a water molecule. Here, it should be noted that the distance of the forming OH bond (1.318 Å, Figure 7) is much shorter than the corresponding distance in IC2 (3.846 Å, Figure 6). This means that the approach of the phosphate moiety to the OH group occurs in the very initial stage of the second step. Apart from this, there are also three important changes in the second step: (i) the proton transfer to H_2_PO_4_^−^, (ii) the C–O bond cleavage, and (iii) the proton transfer from H_2_PO_4_^−^. Change (i) occurs first, and this induces changes (ii) and (iii), which occur rather synchronously. As may be seen by comparing Figure 6 and Figure 7, the H_2_PO_4_^−^·2H_2_O moiety migrates significantly in going from IC2 to TS2, while the skeleton of the main chain and the five-membered ring remains almost unchanged structurally.

Figure 8 shows the resultant product complex (PC), which comprises the succinimide product, a H_2_PO_4_^−^ ion, and three H_2_O molecules. Note that three H_2_O molecules exist in the PC, since a H_2_O molecule has been released. The distance of the broken C–O bond is 3.130 Å (as indicated by boldface in Figure 8). The energy of the PC is lower than that of the RC by about 21 kJ mol^−1^. This may seem to contradict succinimide’s role as an intermediate in the overall reaction scheme, as shown in Scheme 1. However, this is not so. It should be noted that, in the present case, we should use the biochemical conventions for the standard states of water and proton (instead of the physical chemical conventions), because the reactions are postulated to occur in aqueous media near neutral pH [28,34]. For water, the biochemical standard state corresponds to the concentration of 55.5 M. For a reaction that releases a water molecule (such as the reaction studied here), the biochemical standard Gibbs energy of reaction is higher by about 10 kJ mol^−1^ (at 25 °C) than when the physical chemical standard state is used [28]. Therefore, the standard Gibbs energy of reaction is roughly estimated to be about −11 kJ mol^−1^ for the present reaction. This value is consistent with the standard enthalpies and entropies of reaction reported for the Suc formation from two small peptides, Ac–l-Asp–Gly–NHMe and Ac–Gly–l-Asp–Gly–Gly–NHMe [22], from which the standard Gibbs energies of reaction at 25 °C are calculated to be about −5 kJ mol^−1^ (for the protonated form, see below).

There is another factor to be considered. It is usually postulated that in order for the succinimide-forming reactions of Asp residues to occur, the Asp side-chain needs to be protonated, because the Asp γ carbon undergoes a nucleophilic attack. However, at a neutral or physiological pH, the Asp side chains rarely exist in the protonated form. If we use the value of 3.90 for the p*K*_a_ of Asp side chain [28] and the biochemical standard state (pH 7) for the proton, the standard Gibbs energy of the protonated form is calculated to be higher than that of the deprotonated form by about 18 kJ mol^−1^ (at 25 °C). Therefore, roughly speaking, our results show that the standard Gibbs energy of the succinimide product is higher than that of the deprotonated reactant by about 7 kJ mol^−1^. This is consistent with the succinimide being an intermediate.

There was no significant change in the main-chain conformation in the second step (5° in *φ*, and 12° in *ψ*). Throughout the entire reaction from the RC to the PC, there was no significant conformational change on the N-terminal side, as may be seen from the *φ* values reported in Table 1, and the main changes occurred in the C-terminal side and the Asp side chain. This result suggests that the succinimide-forming reaction occurs readily when the C-terminal side of the Asp residue is in a flexible region of proteins, and is also exposed to solvent water. In human αA-crystallin from elderly donors, the Asp58 and Asp151 residues were found to be highly stereoinverted [2]. Interestingly, these two Asp residues were suggested to reside in flexible and solvent-accessible regions [35]. As another example, the X-ray structure of the hen egg-white lysozyme shows that the Asp101 residue, which is prone to succinimide formation, also resides in a flexible and solvent-accessible region, although it is not in an extended conformation, as in the present study [36,37]. Moreover, it would be necessary that the following residue is sterically small, because the catalytic H_2_PO_4_^−^ ion binds to the C-terminal side of the Asp reside (see Figure 3). This is consistent with the well-known notion that the succinimide-mediated reactions mainly occur when the following residue has a sterically small side chain [17,18,35,38,39]. Indeed, the Asp58 and Asp151 residues of human αA-crystallin are followed by serine (Ser) and alanine (Ala) residues, respectively; the Asp101 residue of the hen egg-white lysozyme is followed by a Gly residue.

Very recently, Nakayoshi et al. [30] reported a DFT study on the succinimide formation from Ac–d-Asp–Gly–NHMe catalyzed by a H_2_PO_4_^−^ ion. However, in the reaction pathway they found, an unnaturally large conformational change occurred. On the other hand, in the reaction pathway found in the present study, the structural change of the main-chain and side-chain skeleton is minimized, as may be seen by comparing Figure 3, Figure 4, Figure 5, Figure 6, Figure 7 and Figure 8, and from the dihedral angles listed in Table 1. This may be one of the reasons why our activation barrier (100.3 kJ mol^−1^) is lower than that calculated by Nakayoshi et al. (112.5 kJ mol^−1^) [30].

## 3. Computational Details 

The model compound used in the present study (Ac–l-Asp–NHMe) is shown in Figure 1. A H_2_PO_4_^−^ ion (as a catalyst) and two H_2_O molecules (as solvent molecules stabilizing the negative charge) were included in the calculations. All of the calculations were performed by DFT with the B3LYP functional using Spartan ’14 [40]. Geometry optimizations and vibrational frequency calculations were performed in the gas phase using the 6-31+G(d,p) basis set. All of the optimized geometries were confirmed to be an energy minimum (with no imaginary frequency) or a transition state (with a single imaginary frequency) by vibrational frequency calculations. The energy minima connected by each transition state were confirmed by IRC calculations, followed by full geometry optimizations. Furthermore, single-point calculations of hydration Gibbs energies were carried out for the gas-phase optimized geometries by the SM8 continuum model [32,33], as implemented in Spartan ’14. This solvation model uses partial atomic changes, which can be strongly basis-set dependent. We used the 6-31G(d) basis set for the SM8 calculations, since it is recommended by the developers of the model for giving stable partial atomic changes [41]. The relative energies reported in the present paper include the ZPE and thermodynamic corrections to give the Gibbs energy at 298.15 K, and the SM8 hydration Gibbs energy correction.

## 4. Conclusions

In the present study, we computationally revealed a phosphate-catalyzed mechanism for the succinimide formation from Asp residues. This mechanism proceeds in two steps (cyclization–dehydration); in both, a H_2_PO_4_^−^ ion plays an important role as a proton relay mediator by receiving and donating protons. In both steps, a proton abstraction by the H_2_PO_4_^−^ ion occurs in an early stage, and this induces the subsequent bond reorganization. In particular, the changes in the first step was found to proceed in a domino-like manner. This type of mechanism will be difficult to reveal without calculating the transition state and the IRC. The transition states of both steps have a H_3_PO_4_-like moiety.

The rate-determining step of the succinimide formation from Asp residues was predicted to be the first step, i.e., the cyclization that forms the tetrahedral intermediate. The activation barrier was calculated to be 100.3 kJ mol^−1^ for our model compound, which is in reasonable agreement with an experimental barrier (107 kJ mol^−1^) for the succinimide-forming reaction of an Asp-containing peptide in a phosphate buffer (pH 7.4) [19]. This supports the catalytic mechanism by a H_2_PO_4_^−^ ion, as revealed in the present study. Therefore, there is enough of a possibility that this ion is involved in the succinimide formation from Asp residues in vivo as a catalyst.

In this study, a useful knowledge for understanding age-related changes and diseases was obtained. We used the simplest model compound, but the succinimide-forming reactions are known to be largely affected by the nature (especially the size) of the neighboring residue on the carboxyl side [17,18,35,38,39]. So, further studies are needed for the present mechanism by clarifying the influence of adjacent residues.

Finally, it is interesting to note that a relationship between inorganic phosphate and aging has recently attracted interest in a different field, that of life sciences [42,43,44]. It has been revealed that excess dietary phosphate intake accelerates aging and renal dysfunction [45]. Since the circulating phosphate levels are regulated in a complex manner, further experimental studies are expected to reveal a possible relationship between aging, inorganic phosphate, and the succinimide-mediated non-enzymatic reactions of Asp residues.

## Figures and Tables

**Table 1:** The main-chain dihedral angles *φ* and *ψ*, and the side-chain dihedral angle *χ*_1_ (in degrees) of the optimized geometries ^1^.

Geometry	*φ*	*ψ*	*χ*_1_
RC	−166	177	75
TS1	−166	−145	104
IC1	180	−138	145
IC2	−169	−146	148
TS2	−169	−143	144
PC	−174	−134	128

^1^ See Figure 1 for definitions of *φ*, *ψ*, and *χ*_1_.

## Supplementary Materials

Supplementary materials can be found at http://www.mdpi.com/1422-0067/19/2/637/s1.
