# Supplementary material for: Phosphate-Catalyzed Succinimide Formation from Asp Residues: A Computational Study of the Mechanism
Source: Int J Mol Sci. 2018 Feb 24;19(2):637. doi: 10.3390/ijms19020637 (PMC5855859; doi:10.3390/ijms19020637)
Supplement: Supplementary file 1 [file ijms-19-00637-s001.docx]

Supplementary Materials: Phosphate-Catalyzed Succinimide Formation from Asp Residues: A Computational Study of the Mechanism

Ryota Kirikoshi, Noriyoshi Manabe and Ohgi Takahashi

**Table S1.** Total energies (au), zero-point energies (kJ mol^−1^), and SM8 hydration Gibbs energies (kJ mol^−1^) of the B3LYP/6-31+G(d,p) optimized geometries.

| **Geometry** | **Total energy** | **Zero-point energy** | **SM8 hydration Gibbs energy (6-31G(d))** |
| --- | --- | --- | --- |
| RC | −1481.073869 | 765.7038 | −284.9905425 |
| TS1 | −1481.039183 | 756.3727 | −267.7623427 |
| IC1 | −1481.064199 | 767.3792 | −291.0887940 |
| IC2 | −1481.068226 | 767.4113 | −281.5189515 |
| TS2 | −1481.048043 | 753.8142 | −275.3575414 |
| PC | −1481.070573 | 759.9102 | −307.3469643 |

**Table S2.** Cartesian coordinates (Å) of the reactant complex (RC).

| **Atom** | ***x*** | ***y*** | ***z*** |
| --- | --- | --- | --- |
| 6 | 1.855418700 | −0.144259477 | 1.884547663 |
| 6 | 0.862506711 | 0.083382408 | 0.734358779 |
| 6 | 1.224594369 | 1.183379009 | −0.287230600 |
| 8 | 0.400594001 | 1.553379806 | −1.132684213 |
| 7 | 2.450701802 | 1.750339723 | −0.182078617 |
| 6 | 2.875902951 | 2.795628013 | −1.096290403 |
| 1 | 3.054331982 | 2.406857699 | −2.107283728 |
| 1 | 3.798179523 | 3.242935662 | −0.716883827 |
| 7 | 0.454545282 | −1.141467566 | 0.029443151 |
| 6 | 1.267724096 | −2.060576541 | −0.514325386 |
| 8 | 2.514242761 | −2.056167427 | −0.413098088 |
| 6 | 0.581559207 | −3.168459414 | −1.293524033 |
| 1 | 1.494954552 | −0.998042514 | 2.474016897 |
| 1 | 0.952906562 | −4.132667086 | −0.935132204 |
| 1 | −0.506684394 | −3.134445314 | −1.208615791 |
| 1 | 0.862915776 | −3.075218972 | −2.347586061 |
| 1 | 1.836935576 | 0.737693559 | 2.532772523 |
| 15 | −2.961391081 | 0.326263653 | 0.270778612 |
| 8 | −2.518565152 | 1.256736827 | −1.013844103 |
| 1 | −1.550008637 | 1.392769452 | −1.029224301 |
| 1 | 2.102690728 | 3.564957316 | −1.167400170 |
| 8 | −2.057234361 | 0.620987571 | 1.446744212 |
| 1 | −0.078939648 | 0.435434287 | 1.191400944 |
| 1 | 3.132652848 | 1.259948303 | 0.394487286 |
| 8 | 3.220749232 | −0.333844509 | 1.502377651 |
| 1 | −0.554634817 | −1.239901713 | −0.124387822 |
| 1 | 3.197891956 | −1.037427126 | 0.808364984 |
| 8 | −4.460007841 | 0.275537039 | 0.314295709 |
| 8 | −2.422470153 | −1.211401831 | −0.259778007 |
| 1 | −3.083759466 | −1.555337246 | −0.875081055 |

**Table S3.** Cartesian coordinates (Å) of the transition state TS1.

| **Atom** | ***x*** | ***y*** | ***z*** |
| --- | --- | --- | --- |
| 6 | −1.862508679 | −0.058242986 | 1.728822258 |
| 6 | −0.920816313 | −0.056812340 | 0.559039572 |
| 6 | −0.960973320 | −1.137652260 | −0.366999483 |
| 8 | −0.195409381 | −1.255947207 | −1.398156541 |
| 7 | −1.827549866 | −2.175433854 | −0.107868521 |
| 6 | −1.868291143 | −3.356041913 | −0.949347921 |
| 1 | −2.330171818 | −3.167464490 | -1.929873655 |
| 1 | −2.437978495 | −4.137475851 | −0.435657765 |
| 7 | −0.570301509 | 1.242079308 | 0.015571905 |
| 6 | −1.466810705 | 2.129167054 | −0.469190628 |
| 8 | −2.700016464 | 1.959664260 | −0.457397168 |
| 6 | −0.885003626 | 3.417817354 | −1.033323744 |
| 1 | −1.664750891 | 0.826145029 | 2.350513151 |
| 1 | −1.260740692 | 4.265063307 | −0.450296614 |
| 1 | 0.208278357 | 3.432391749 | −1.033410956 |
| 1 | −1.245941446 | 3.537596957 | −2.059102394 |
| 1 | −1.710202881 | −0.946870944 | 2.352323354 |
| 15 | 2.752449461 | −0.171221044 | 0.155481299 |
| 8 | 2.122074991 | −0.570974539 | −1.210501802 |
| 1 | 1.031703047 | −0.843398709 | −1.289646369 |
| 1 | −0.852838626 | −3.716266971 | −1.127860995 |
| 8 | 1.812890407 | −0.728281921 | 1.342461299 |
| 1 | 0.826476072 | −0.567388156 | 1.175752708 |
| 1 | −2.675851574 | −1.900922558 | 0.376889990 |
| 8 | −3.279421067 | −0.097466588 | 1.386446527 |
| 1 | 0.405623222 | 1.522073888 | −0.010371260 |
| 1 | −3.373607719 | 0.588464742 | 0.693458853 |
| 8 | 4.194025575 | −0.446395027 | 0.408500446 |
| 8 | 2.463697339 | 1.477063886 | 0.181857741 |
| 1 | 3.021912667 | 1.879137134 | 0.861454437 |

**Table S4.** Cartesian coordinates (Å) of the tetrahedral intermediate complex IC1.

| **Atom** | ***x*** | ***y*** | ***z*** |
| --- | --- | --- | --- |
| 6 | −1.963559929 | 0.067819071 | 1.652393389 |
| 6 | −1.050820656 | −0.021667774 | 0.463841659 |
| 6 | −1.109526162 | −1.140775956 | −0.355418736 |
| 8 | −0.347978309 | −1.365190455 | −1.418071577 |
| 7 | −2.006814343 | −2.144593135 | −0.070152664 |
| 6 | −2.046518367 | −3.382719934 | −0.827175686 |
| 1 | −2.456916993 | −3.256206028 | −1.839687061 |
| 1 | −2.665161241 | −4.104317203 | −0.284206921 |
| 7 | −0.510026495 | 1.205210099 | −0.060074264 |
| 6 | −1.290780984 | 2.228793068 | −0.474402753 |
| 8 | −2.534836456 | 2.217925992 | −0.429593883 |
| 6 | −0.552746012 | 3.449463367 | −1.000696290 |
| 1 | −1.689695393 | 0.942985050 | 2.256891923 |
| 1 | −0.811174032 | 4.316132957 | −0.383594320 |
| 1 | 0.532529835 | 3.320542949 | −1.012685589 |
| 1 | −0.904146912 | 3.652164537 | −2.016848048 |
| 1 | −1.871392990 | −0.822721755 | 2.284526735 |
| 15 | 2.791120961 | −0.268002018 | 0.116188293 |
| 8 | 2.016626863 | −0.591419270 | −1.156270692 |
| 1 | 0.614208672 | −0.977841478 | −1.320739370 |

**Table S4.** *Cont.*

| **Atom** | ***x*** | ***y*** | ***z*** |
| --- | --- | --- | --- |
| 1 | −1.038197689 | −3.789982614 | −0.926439197 |
| 8 | 1.965409423 | −0.878545565 | 1.405500089 |
| 1 | 1.000317906 | −0.778449134 | 1.274012112 |
| 1 | −2.871041943 | −1.812302691 | 0.342475626 |
| 8 | −3.374945984 | 0.132915736 | 1.313997137 |
| 1 | 0.496399967 | 1.351110709 | −0.081468155 |
| 1 | −3.419040626 | 0.844693263 | 0.643512622 |
| 8 | 4.245860083 | −0.551102190 | 0.301165688 |
| 8 | 2.509611789 | 1.382768590 | 0.294090465 |
| 1 | 3.080116287 | 1.725609188 | 0.995226423 |

**Table S5.** Cartesian coordinates (Å) of the tetrahedral intermediate complex IC2.

| **Atom** | ***x*** | ***y*** | ***z*** |
| --- | --- | --- | --- |
| 6 | 1.776772672 | −0.119314343 | 1.879104488 |
| 6 | 0.865880902 | 0.108522277 | 0.664814053 |
| 6 | 1.293208967 | 1.201712888 | −0.338537243 |
| 8 | 0.526640938 | 1.549187915 | −1.245728908 |
| 7 | 2.506532462 | 1.776197900 | −0.150148459 |
| 6 | 2.998519442 | 2.808758278 | −1.044673617 |
| 1 | 3.327725510 | 2.396495375 | −2.007978697 |
| 1 | 3.840447990 | 3.319476497 | −0.569748477 |
| 7 | 0.502632297 | −1.111167830 | −0.073958416 |
| 6 | 1.322242945 | −2.088010869 | −0.494545139 |
| 8 | 2.538957378 | −2.166731470 | −0.217176283 |
| 6 | 0.673480298 | −3.165966466 | −1.344776286 |
| 1 | 1.374920127 | −0.969604413 | 2.446058230 |
| 1 | 0.804936174 | −4.130727338 | −0.845421973 |
| 1 | −0.390202455 | −2.991644237 | −1.521299230 |
| 1 | 1.199738175 | −3.215079555 | −2.302916141 |
| 1 | 1.714343915 | 0.764664561 | 2.521357963 |
| 15 | −2.946986148 | 0.164974979 | 0.278623933 |
| 8 | −3.739304563 | −1.108816753 | 0.392519073 |
| 8 | −1.962491174 | −0.033097318 | −1.068710797 |
| 1 | −1.354103683 | 0.723425429 | −1.187250631 |
| 8 | −2.084248253 | 0.743016560 | 1.376339540 |
| 1 | −0.099293892 | 0.464564766 | 1.068007376 |
| 1 | 3.147298908 | 1.285173791 | 0.472289319 |
| 8 | 3.164001536 | −0.311201868 | 1.590646692 |
| 1 | −0.453730106 | −1.086740909 | −0.438453123 |
| 1 | 3.181956717 | −1.057799827 | 0.943057093 |
| 8 | −3.970184404 | 1.379943079 | −0.230178667 |
| 1 | 2.201992068 | 3.529351081 | −1.244259325 |
| 1 | −4.722993566 | 0.945997864 | −0.654055985 |

**Table S6.** Cartesian coordinates (Å) of the transition state TS2.

| **Atom** | ***x*** | ***y*** | ***z*** |
| --- | --- | --- | --- |
| 6 | −1.851171010 | −0.369214207 | 1.688307930 |
| 6 | −0.965362749 | −0.130348872 | 0.501840735 |
| 6 | −0.722303169 | −1.182022080 | −0.426926295 |
| 8 | 0.048038758 | −1.071630476 | −1.454196282 |
| 7 | −1.269672336 | −2.418102669 | −0.166602604 |
| 6 | −0.994977916 | −3.559899215 | −1.017682430 |
| 1 | −1.515601385 | −3.508159989 | −1.985687551 |
| 1 | −1.305571299 | −4.473075777 | −0.499203307 |
| 7 | −0.940207701 | 1.210302103 | −0.050383307 |
| 6 | −2.029534584 | 1.921213741 | −0.412535018 |
| 8 | −3.204726430 | 1.549276572 | −0.232123810 |
| 6 | −1.754353550 | 3.280340703 | −1.039808414 |
| 1 | −1.859565132 | 0.530126533 | 2.318951153 |
| 1 | −2.165013992 | 4.062206701 | −0.392554035 |
| 1 | −0.690454460 | 3.474140294 | −1.202694992 |
| 1 | −2.281784250 | 3.333213229 | −1.996846679 |
| 1 | −1.465161890 | −1.197899146 | 2.292539361 |
| 15 | 2.717878491 | 0.385088858 | 0.228906480 |
| 8 | 3.640440160 | 1.518543531 | 0.534764033 |
| 8 | 1.783418318 | 0.600207654 | −1.027002584 |
| 1 | 1.025754391 | −0.168422976 | −1.262738695 |
| 8 | 1.762560999 | −0.079122254 | 1.428568431 |
| 1 | 0.778479156 | −0.165244125 | 1.172825842 |
| 1 | −2.158215916 | −2.386603638 | 0.321673369 |
| 8 | −3.220615898 | −0.756627898 | 1.380483733 |
| 1 | −0.035826734 | 1.577572732 | −0.328850719 |
| 1 | −3.520677961 | −0.063067341 | 0.758411295 |
| 8 | 3.592685636 | −0.982302422 | −0.061848554 |
| 1 | 4.510025165 | −0.709582232 | −0.197387692 |
| 1 | 0.076402715 | −3.614111247 | −1.221471956 |

**Table S7.** Cartesian coordinates (Å) of the product complex (PC).

| **Atom** | ***x*** | ***y*** | ***z*** |
| --- | --- | --- | --- |
| 6 | −2.405609832 | −0.129333753 | 1.463336330 |
| 6 | −1.380567717 | −0.117325923 | 0.370698852 |
| 6 | −1.056926179 | −1.263574419 | −0.308830476 |
| 8 | −0.110003233 | −1.371687290 | −1.253361215 |
| 7 | −1.722578217 | −2.455215201 | −0.042605677 |
| 6 | −1.371913495 | −3.678624180 | −0.745803804 |
| 1 | −1.603586454 | −3.648838593 | −1.820993572 |
| 1 | −1.922617674 | −4.507428900 | −0.289396519 |
| 7 | −0.880373856 | 1.143638474 | −0.088071695 |
| 6 | −1.677397650 | 2.177575663 | −0.433438020 |
| 8 | −2.921187336 | 2.162709263 | −0.340222324 |
| 6 | −0.958906870 | 3.423349848 | −0.925415620 |
| 1 | −2.264943375 | 0.755103391 | 2.099140741 |
| 1 | −1.099062179 | 4.228550703 | −0.196125352 |
| 1 | 0.111481348 | 3.265959688 | −1.081057018 |
| 1 | −1.423839321 | 3.743316868 | −1.862363566 |
| 1 | −2.285213472 | −1.020694361 | 2.088869502 |

**Table S7.** *Cont.*

| **Atom** | ***x*** | ***y*** | ***z*** |
| --- | --- | --- | --- |
| 15 | 2.917590379 | 0.217663269 | 0.168369181 |
| 8 | 4.023444040 | 1.219056605 | 0.275466614 |
| 8 | 1.713974730 | 0.399386397 | −0.764069008 |
| 1 | 0.631281036 | −0.692431271 | −1.124794958 |
| 8 | 2.296982833 | −0.049275706 | 1.678323191 |
| 1 | 1.393012965 | −0.392410589 | 1.612188676 |
| 1 | −2.708682435 | −2.321082108 | 0.140688148 |
| 8 | −3.773020212 | −0.180103460 | 0.995083169 |
| 1 | 0.124679113 | 1.204794248 | −0.282242925 |
| 1 | −3.830796787 | 0.590886828 | 0.395390360 |
| 8 | 3.551469106 | −1.260757423 | −0.235714325 |
| 1 | −0.302357597 | −3.874092706 | −0.643340464 |
| 1 | 4.492104703 | −1.242575646 | −0.014091557 |


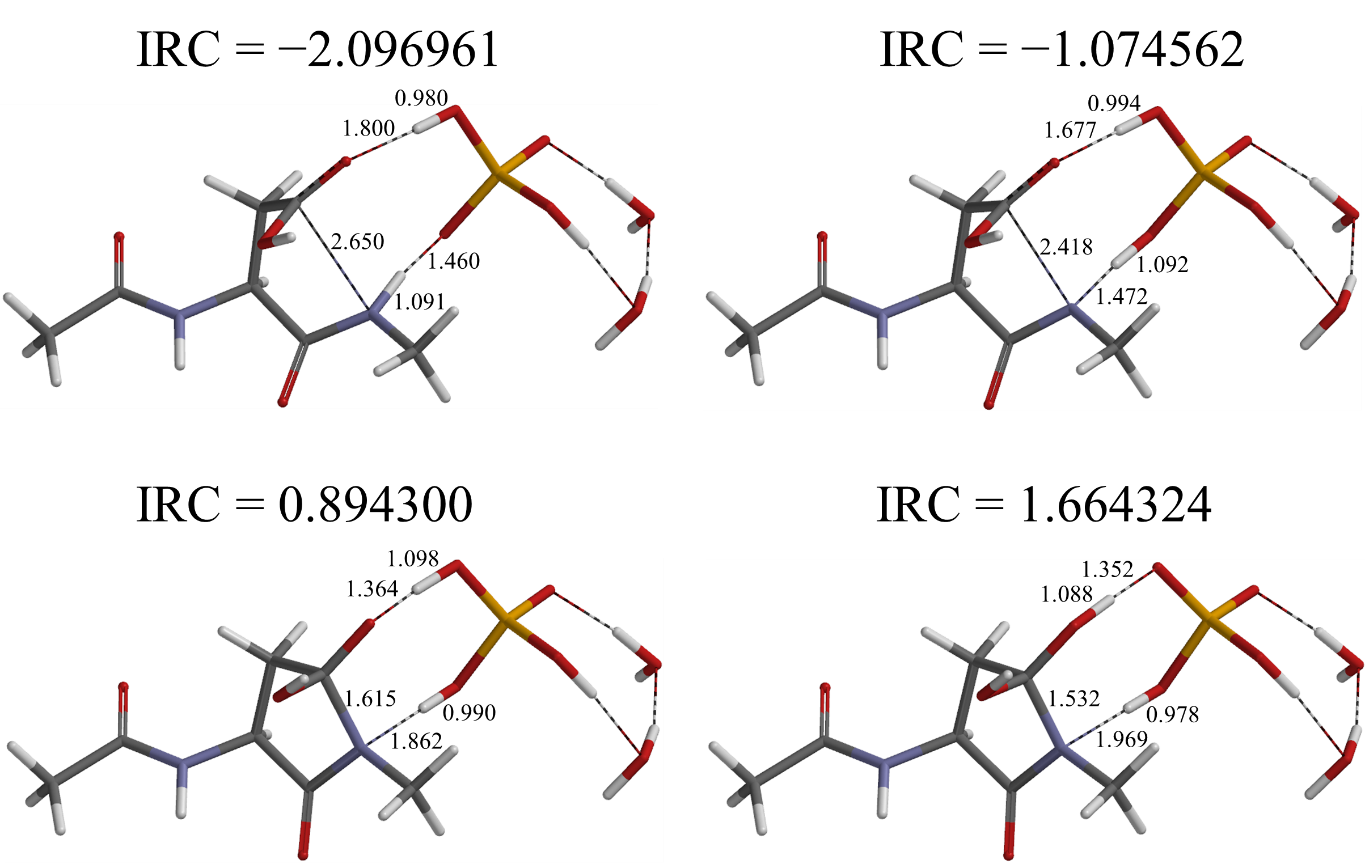


**Figure S1.** Four representative geometries on the intrinsic reaction coordinate (IRC) of the first step, showing the domino-like changes in this step. The IRC values are in mass-weighted atomic units. Relevant interatomic distances are shown in Å.
